# The Time is Now to Control Typhoid

**DOI:** 10.1093/cid/ciy1115

**Published:** 2019-03-07

**Authors:** Andrew J Pollard, Anthony A Marfin, Kathleen M Neuzil

**Affiliations:** 1 Oxford Vaccine Group, University of Oxford, and the National Institute for Health Research Oxford Biomedical Research Centre, United Kingdom; 2 Center for Vaccine Innovation and Access, PATH, Seattle, Washington; 3 Center for Vaccine Development and Global Health at the University of Maryland School of Medicine, Baltimore, MD

**Keywords:** TyVAC, typhoid, vaccine

The death of Prince Albert, married to Queen Victoria of the United Kingdom, in 1861 from typhoid profoundly impacted the Monarchy. The queen mourned for the rest of her life, some 40 years, highlighting the individual and familial impact of typhoid in a very public way [1]. The diagnosis was made by the prince’s physician, Sir William Jenner [2], who managed many typhoid patients and meticulously documented the prince’s symptoms, including the appearance of rose spots.

As a result of municipal clean water and sewage works, typhoid has essentially disappeared from the wealthy nations of the world, with almost all cases in Europe and North America now attributed to travel to endemic regions. However, there are still thought to be nearly 12 million cases and more than 128 000 deaths each year associated with *Salmonella* serotype Typhi [3]. Cases occur largely in resource-poor regions of the world, sub-Saharan Africa, and South/Southeast Asia, where there is limited access to clean water and inadequate sanitation [4, 5]. In these communities, the disease continues to have the same profound impact on families as was experienced by Queen Victoria. Fortunately, with the availability of antimicrobial therapy, the mortality from typhoid has fallen from the high levels of the preantibiotic era. Recently, however, extensively drug-resistant (XDR) *S. typhi* variants have emerged in India, Bangladesh, the Philippines, Iraq, and Guatemala, and, importantly, have caused a large outbreak in Pakistan [6, 7], further threatening the health of these populations.

Since 2008, the World Health Organization (WHO) has advocated vaccine control of typhoid [8], but uptake has been slow due to lack of a suitable vaccine for children aged <2 years. Of course, typhoid can be controlled by use of clean water and improved sanitation and hygiene, which would stop the spread of typhoid and other water- and foodborne enteric pathogens. However, we cannot wait for safe water and proper sanitation to be available for every child; too many will suffer from enteric fever if we do not act. It is time to control typhoid now.

The Typhoid Vaccine Acceleration Consortium (TyVAC) [9], funded by the Bill & Melinda Gates Foundation, launched in 2017 as a partnership of the Center for Vaccine Development and Global Health at the University of Maryland School of Medicine, the Oxford Vaccine Group at the University of Oxford, and PATH, an international nonprofit organization. TyVAC’s mission is to protect children who suffer the greatest burden by accelerating the use of typhoid conjugate vaccine (TCV) in Africa and Asia. As a major component, the consortium is evaluating Typbar-TCV, the leading vaccine candidate, to provide data to support introduction of the vaccine in those regions suffering a substantial burden of disease. The studies were designed to be complementary to each other and to other efforts, including data available from the vaccine manufacturer and partners [10].

This supplement highlights some of the current and future work of TyVAC and its associates. The approach is similar to that used to help drive global introductions of other vaccines, as summarized by Jamka et al [11]. With the March 2018 WHO-revised typhoid position paper and the current Gavi, the Vaccine Alliance, funding window for TCV introduction, the supplement provides key data on vaccine efficacy and immunogenicity in sub-Saharan Africa and Southeast Asia. The supplement includes methods papers for the 4 TyVAC sites and articles on health economics, modeling, demand forecasting, global incidence, public engagement and lessons learned, antimicrobial resistance, decision-making, data management, and accelerating TCV introduction.

The supplement includes a systematic review of data on the global burden of typhoid [12] and a series of important articles on new burden data from Myanmar, Democratic Republic of Congo, Bangladesh, and Tanzania [13–16]. As identified in these articles, and others published recently [17, 18], typhoid is far more widespread than might be appreciated from the microbiological literature. Surveillance for the disease requires microbiological culture facilities since the clinical syndrome is largely indistinguishable from other illnesses that present with fever, making syndromic surveillance unhelpful. Unfortunately, the regions with the highest rates of typhoid often have the least developed laboratory facilities, emphasizing the importance of these studies in supporting vaccine deployment and control of the disease. Lack of country- and regional-level surveillance data is one of the main challenges for decision-makers.

Vaccine efficacy of 54%–87% was recently demonstrated using a controlled human infection model of typhoid [19], which strongly supports the likely efficacy of Typbar-TCV, manufactured by Bharat Biotech. The study was run using human adult volunteers in Oxford, United Kingdom, who had not lived in an endemic area but received 10 000 bacteria as the infection challenge after neutralization of gastric acid. It is unclear how efficacy in the model relates to performance of this TCV in children in an endemic region where there are currently no field efficacy data. Liu et al [20] discuss the design and analysis of seroefficacy studies for TCVs.

TCV was licensed and recommended by WHO based on immunogenicity data and a human controlled infection model in nonendemic adults, but the lack of field data at this time undermines confidence at the country level of the potential impact. Given limitations on health budgets, many countries will need more information before deciding to introduce a vaccine, particularly data are needed on impact, how programs might be structured, and how the vaccine performs in different transmission settings. As part of the TyVAC program, we are undertaking individually randomized clinical trials in Malawi and Nepal and a cluster-randomized trial in Bangladesh. The protocols for these studies are summarized in this supplement, in addition to an immunogenicity trial protocol from Burkina Faso [21–24]. In the efficacy trials, more than 85 000 children from age 9 months to age 15 years were vaccinated during late 2017 through the first half of 2018. Given that duration of immunity is critically important to country-level decisions on vaccine introduction, these children will be followed for at least 2 years using enhanced passive microbiological surveillance (blood cultures), with vaccine efficacy measures available by 2020. The control vaccine in these studies is either a capsular group A meningococcal vaccine (Nepal and Malawi), as this is the main agent causing meningococcal disease in these regions, or Japanese encephalitis vaccine in Bangladesh, since this disease is important in rural areas of that country. Given the importance of typhoid in the population, control-arm children in Nepal and Bangladesh will receive TCV at the end of the study. In Malawi, the epidemiology and burden are less certain; therefore, the decision on whether or not to provide TCV for children in the control group will be made in concert with Malawian health officials once data are available. Other typhoid vaccines were not considered for control vaccines because they are not licensed for the youngest children, do not confer long-term protection, and, therefore, are less suitable for routine country introduction. Another important outstanding question is the potential role of TCV in reducing transmission of typhoid in the field and thus inducing herd protection, as was recently indicated as possible in studies using the human challenge model [25]. The TyVAC field trials will directly address this through the cluster-randomized trial in Bangladesh. Taken together, the data from the TyVAC trials will provide valuable information to inform vaccine deployment in Africa and Asia, and we anticipate reassurance on field safety will be important for national immunization technical advisory groups (NITAGs) and health ministers, when considering introduction of new TCVs. The process of obtaining funding for introductions, especially through international donors, is slow. The TyVAC trials will provide important data ahead of possible introductions in most countries [26].

Important components of vaccine field studies that are rarely described in the scientific literature are logistical elements of study delivery and the role and implementation of public engagement. Two articles in this supplement deal with these issues and provide a high-level view that we anticipate will be useful for deployment of clinical studies in the future [27, 28].

As mentioned above, antimicrobial resistance is a major threat to the health of populations in which Salmonella infections are prevalent as these bacteria readily pick up resistance genes. Multidrug resistance (MDR, defined as resistance to the first-line antibiotics ampicillin, cotrimoxazole, and chloramphenicol) was recognized several decades ago and became widespread among the agents of enteric fever. However, there has been a decline in resistance over the past 10 years, especially in South Asia, as antibiotic pressure was reduced after these drugs became redundant in typhoid treatment [29]. More recent use of fluoroquinolones has driven very widespread acquisition of resistance to this class of antibiotic; currently severe cases often must be treated with cephalosporins and milder cases with azithromycin. A recent outbreak of XDR typhoid (defined as MDR plus resistance to fluoroquinolones and cephalosporins) has been identified in Pakistan [7], considerably reducing treatment options. In this supplement, we provide insight into antimicrobial resistance (AMR) from genomic analyses of typhoid strains [30] and country-specific data on AMR in the enteric fever burden studies. Introduction of new TCVs will impact AMR infections in the same way as susceptible typhoid, and our modeling article summarizes predicted vaccine impact [31].

In October 2017, TyVAC provided evidence for WHO’s Strategic Advisory Group of Experts (SAGE) on Immunization that recommended the use of new TCVs. Also, WHO supplied information for Gavi, the Vaccine Alliance, to support Gavi’s planning for TCV financing, with $85 million provided for vaccine deployment as outlined by Jamka et al [11]. An important component of country decision-making for new vaccines, especially for those graduating from Gavi support for new vaccine programs in the next 5 years, is the economics of vaccine introduction. In this supplement, we include a review of typhoid vaccine economic studies and summarize plans for further work of the TyVAC consortium [32]. For manufacturers and vaccine financiers (Gavi), an accurate demand forecast is important to ensure supply meets demand and funds are available to purchase products. Reducing uncertainty is important for country-level decisions, as highlighted by supply shortages for other vaccines in recent years, which have caused difficulties for national programs. The demand-forecasting article describes the model built to estimate TCV demand [33].

Finally, in this supplement, we discuss the importance of communications about typhoid, which must involve international, national, and local-level stakeholders. This ensures that SAGE recommendations are translated into appropriate decisions by the NITAG, with government support, and are clear and understood by local public health immunization officials and frontline staff so that new programs are introduced rapidly and with clarity for the benefit of the population [34].

The articles in this supplement show the development of high-quality evidence by TyVAC to inform decision-making on TCV introduction. Through the data produced and the advocacy of TyVAC, we are tasked with improving health among some of the most vulnerable populations in the world through introduction of TCVs where they are most needed to drive substantial and clinically meaningful reductions in typhoid burden. There should be no more families to mourn like Queen Victoria did. It is time to control typhoid.

## Notes

***Financial support.*** This publication is based on research funded in part by a grant from the Bill & Melinda Gates Foundation (OPP1151153).

***Supplement sponsorship.*** This supplement is sponsored by the Center for Vaccine Development and Global Health (CVD) at the University of Maryland School of Medicine.

***Potential conflicts of interest.*** A. J. P. reports grants from Okairos outside the submitted work; is chair of UK Department of Health’s Joint Committee on Vaccination and Immunisation and the EMA scientific advisory group on vaccines; and is a member of the World Health Organization’s Strategic Advisory Group of Experts on Immunization. K. M. N. is a member of the World Health Organization Strategic Advisory Group of Experts on Immunization. All other authors reported no potential conflicts. The authors have submitted the ICMJE Form for Disclosure of Potential Conflicts of Interest. Conflicts that the editors consider relevant to the content of the manuscript have been disclosed.
